# Supplementary material for: Detection of relativistic fermions in Weyl semimetal TaAs by magnetostriction measurements
Source: Nat Commun. 2022 Jul 5;13:3868. doi: 10.1038/s41467-022-31321-4 (PMC9256615; doi:10.1038/s41467-022-31321-4)
Supplement: Supplementary file 1 — Supplementary Information [file 41467_2022_31321_MOESM1_ESM.pdf]

Supplementary Information

Detection of relativistic fermions in Weyl semimetal TaAs by magnetostriction measurements

Tomasz Cichorek, Łukasz Bochenek, and Jarosław Juraszek

Institute of Low Temperature and Structure Research, Polish Academy of Sciences, 50-422 Wrocław, Poland

Yuriy V. Sharlai and Grigorii P. Mikitik

B. Verkin Institute for Low Temperature Physics and Engineering, Ukrainian Academy of Sciences, Kharkov 61103, Ukraine

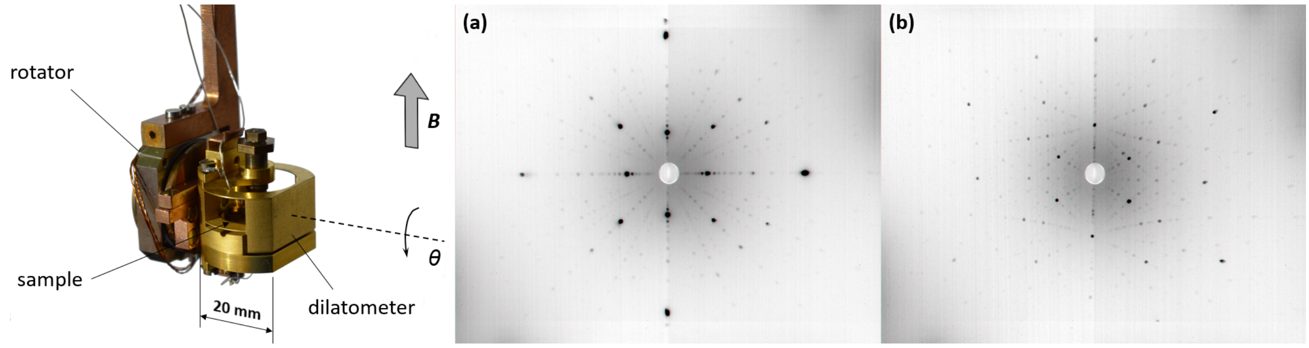

Supplementary Fig. 1. Left: Photograph of the commercial dilatometer cell [1] mounted on a cold finger of a dilution refrigerator. This setup is equipped with a piezoelectric rotator for angle-dependent magnetostriction. Right: Back-reflection Laue photographs of the TaAs single crystal (sample 1). Figure (a) shows the diffractogram along the c axis, whereas (b) shows the diffractogram along the a axis.

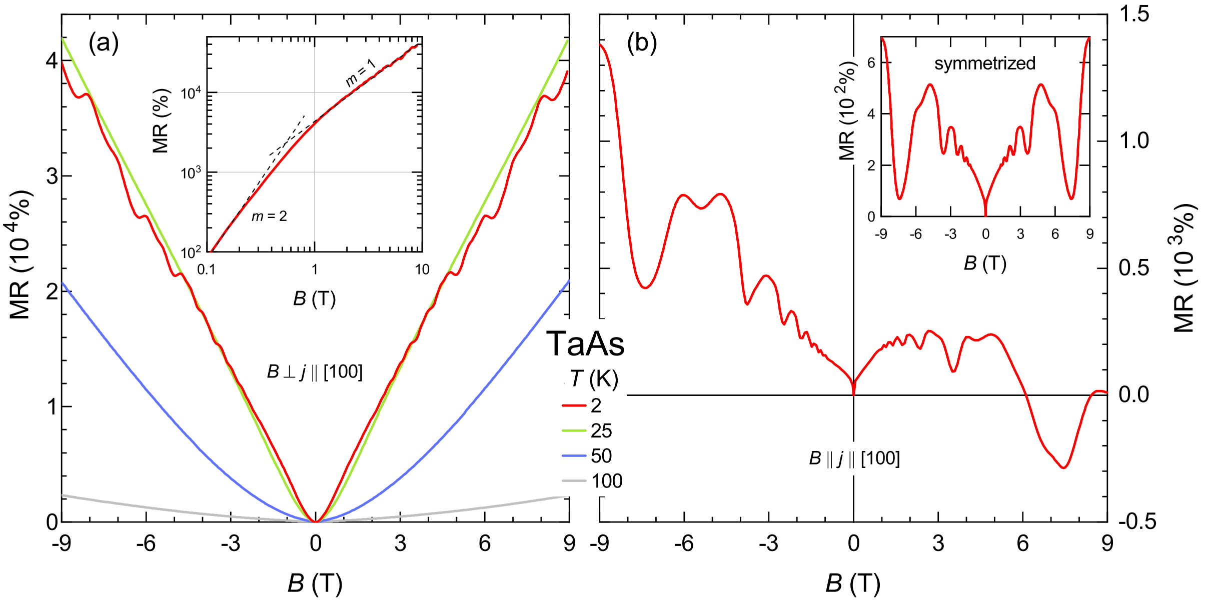

Supplementary Fig. 2. Transverse (a) and longitudinal (b) magnetoresistance of TaAs measured along the a axis. No off-diagonal components were subtracted from the MR data shown in main panels.

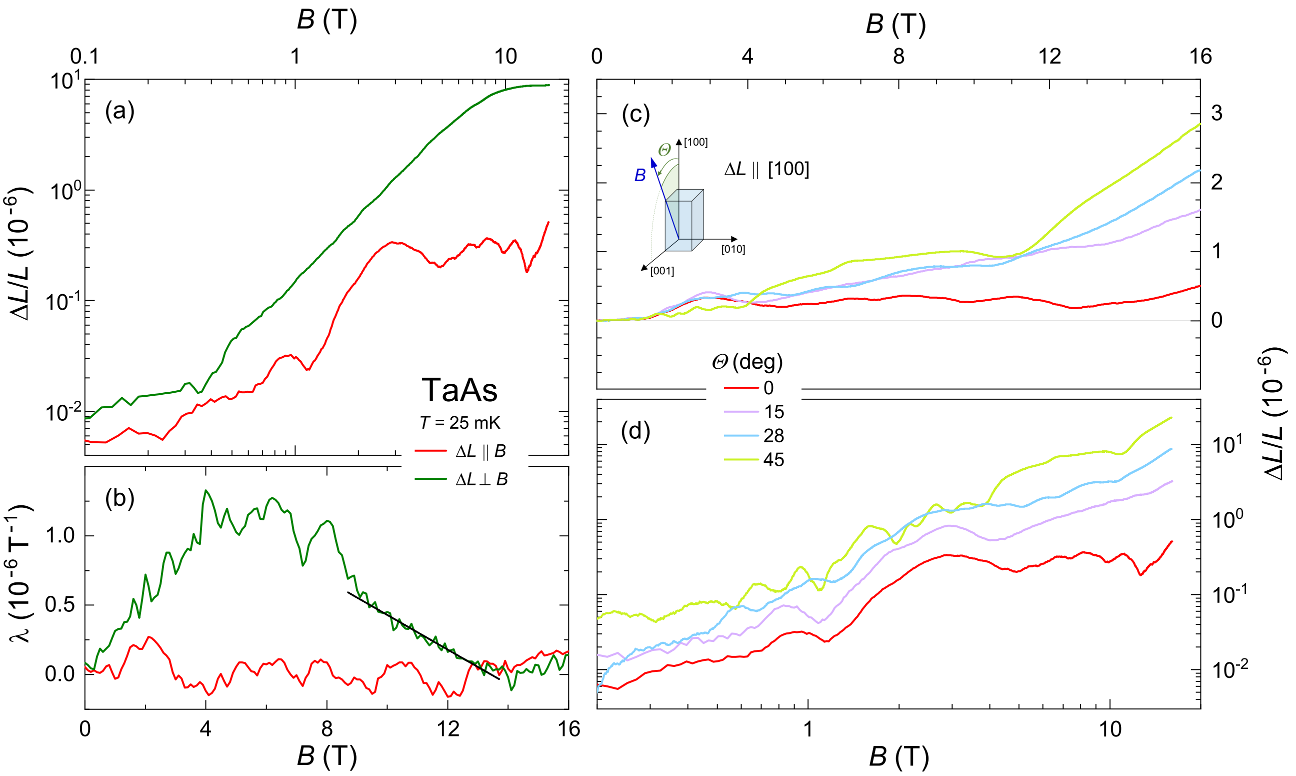

Supplementary Fig. 3. The a-axis magnetostriction of TaAs measured within the (010) plane at 25 mK. (a) Magnetic-field dependence of the relative length change $\Delta L/L$ measured along the [100] direction both in the parallel (red) and perpendicular (green) configuration. At 16 T, the $\Delta L \parallel B$ and $\Delta L \perp B$ data differ by more than one order of magnitude. (b) Magnetostriction coefficient λ derived from the longitudinal and transverse measurements. The black straight line with the slope -12.5×10^{-8} marks the range where λ is a linear function of B. (c) Complex dependences of the magnetostriction at small tilt angles $\Theta \leq 45^{\circ}$. Note a small longitudinal magnetostriction in the entire field range (red). (d) Log-log plot of the experiential results shown in (c). Curves are subsequently shifted by a factor of 3 for clarity.

Supplementary Note 1: Formula for the magnetostriction

We now derive the general formula for the magnetostriction [formula (1) in the main text], assuming, for definiteness, the tetragonal symmetry of a crystal (i.e., the symmetry of TaAs). This formula can be obtained by a minimization of the Ω potential for the deformed crystal placed in the external magnetic field H with respect to the strain tensor u_{ik},

$$\begin{aligned}\Omega = &\frac{1}{2}C_{11}(u_{xx}^2+u_{yy}^2)+\frac{1}{2}C_{33}u_{zz}^2\\ &+C_{13}(u_{xx}+u_{yy})u_{zz}+C_{12}u_{xx}u_{yy}\\ &+2C_{66}u_{xy}^2+2C_{44}(u_{xz}^2+u_{yz}^2)\\ &+\Delta\Omega_{\rm el}(u_{ik},B)-\Delta\Omega_{\rm el}(u_{ik},0), \end{aligned} \quad (1)$$

where C_{mn} are the elastic moduli of the crystal [2], $B=\mu_0 H$ is the magnetic induction in the sample, $\Delta\Omega_{\rm el}(u_{kl},B)\equiv\Omega_{\rm el}(u_{kl},B)-\Omega_{\rm el}(0,B)$, and $\Omega_{\rm el}(u_{kl},B)=\sum_{i=1}^{N}\Omega_i(u_{kl},B)$ is the deformation-dependent part of the electron Ω potential for the crystal with N pockets of charge carriers. This part depends on the deformation, the magnetic induction B, and also on the temperature T and the chemical potential ζ (we do not indicate explicitly T and ζ here). The first six terms in Eq. (1) give the total elastic energy of a deformation. This energy is partly produced by $\Delta\Omega_{\rm el}(u_{kl},0)$, and hence the difference of the elastic terms and $\Delta\Omega_{\rm el}(u_{kl},0)$ is the elastic energy that is not associated with the electron pockets under study. The term $\Delta\Omega_{\rm el}(u_{kl},B)$ describes the total change in the Ω potential of these pockets in the magnetic field under the deformation. As in Ref. [3–6], we assume that an elastic deformation shifts the electron bands as a whole and does not change their shape. This rigid-band approximation commonly is well justified for sufficiently small pockets (see below). In particular, this approximation seems to be valid for TaAs family of the semimetals, see Figs. 6e and 6f in Ref. [7]. In this rigid-band approximation, a shift $\Delta\varepsilon_i$ of the ith electron energy band $\varepsilon_i(\mathbf{p})$ under the deformation coincides with the shift of its edge and is proportional to the strain tensor u_{kl}, $\Delta\varepsilon_i=\sum_{k,l}\lambda_{kl}^{(i)}u_{kl}$, where $\mathbf{p}$ is the electron quasimomentum, and the constants $\lambda_{kl}^{(i)}$ are the so-called deformation potential of the ith band [8]. Therefore, in the minimization of Ω potential, one can use the following relation:

$$\frac{\partial\Omega_i(u_{kl},B)}{\partial u_{kl}}=-\frac{\partial\Omega_i(\zeta,B)}{\partial\zeta}\frac{\partial\Delta\varepsilon_i}{\partial u_{kl}}=\lambda_{kl}n_i(B),$$

where $n_i(B)=-\partial\Omega_i(\zeta,B)/\partial\zeta$ is the density of charge carriers of the pocket i in the magnetic field. The minimization gives the set of the equations in the tensor u_{kl}, which defines the magnetostriction, i.e., the deformation of the crystal in the magnetic field,

$$C_{33}u_{zz}+C_{13}(u_{xx}+u_{yy})=-\sum_i\lambda_{zz}^{(i)}[n_i(B)-n_i(0)],$$
$$C_{11}u_{xx}+C_{13}u_{zz}+C_{12}u_{yy}=-\sum_i\lambda_{xx}^{(i)}[n_i(B)-n_i(0)],$$
$$C_{11}u_{yy}+C_{13}u_{zz}+C_{12}u_{xx}=-\sum_i\lambda_{yy}^{(i)}[n_i(B)-n_i(0)],$$
$$4C_{66}u_{xy}=-\sum_i\lambda_{xy}^{(i)}[n_i(B)-n_i(0)],$$
$$4C_{44}u_{xz}=-\sum_i\lambda_{xz}^{(i)}[n_i(B)-n_i(0)],$$
$$4C_{44}u_{yz}=-\sum_i\lambda_{yz}^{(i)}[n_i(B)-n_i(0)],$$

where $i=1,\dots,N$ usually runs several groups of the equivalent Fermi pockets. (We call pockets equivalent if, in absence of the magnetic field, they transform into each other under symmetry operations.) It follows from symmetry considerations that inside each of the groups, the parameters $\lambda_{zz}^{(i)}$ have one and the same value, the $\lambda_{xx}^{(i)}$ and $\lambda_{yy}^{(i)}$ take on only the two values λ_{xx}^{a1} and λ_{xx}^{a2} with $\lambda_{yy}^{a1}=\lambda_{xx}^{a2}$, $\lambda_{yy}^{a2}=\lambda_{xx}^{a1}$, whereas the off-diagonal $\lambda_{lz}^{(i)}$ (with $l=x,y$) run the four values $\pm\lambda_{lz}^{a1}$, $\pm\lambda_{lz}^{a2}$, and $\lambda_{xy}^{(i)}$ is equal to $\pm\lambda_{xy}$. Solving this set of the equations, we obtain,

$$u_{zz}=\sum_i\Lambda_i[n_i(B)-n_i(0)], \quad (2)$$

$$\Lambda_i=-\frac{\lambda_{zz}^{(i)}(C_{11}+C_{12})-2C_{13}\bar\lambda_{xx}^{(i)}}{C_{33}(C_{11}+C_{12})-2(C_{13})^2}\equiv\Lambda_i^c. \quad (3)$$

The same formula (2) describes $(u_{xx}+u_{yy})/2$ but with the other Λ_i,

$$\Lambda_i=-\frac{\bar\lambda_{xx}^{(i)}C_{33}-\lambda_{zz}^{(i)}C_{13}}{C_{33}(C_{11}+C_{12})-2(C_{13})^2}\equiv\Lambda_i^{\perp}. \quad (4)$$

In Eqs. (3) and (4), $\bar\lambda_{xx}^{(i)}\equiv(\lambda_{xx}^{a1}+\lambda_{xx}^{a2})/2$ for all i belonging to the appropriate group of the equivalent pockets. It is significant that Λ_i^c and $\Lambda_i^{\perp}$ have one and the same values inside each of these groups. On the other hand, the coefficients Λ_i in formula (2), which also describes u_{xx} and u_{yy}, take on the two different values for the equivalent Fermi pockets:

$$\Lambda_i^{a1}=\Lambda_i^{\perp}-\frac{\lambda_{xx}^{a1}-\lambda_{xx}^{a2}}{2(C_{11}-C_{12})},\quad \Lambda_i^{a2}=\Lambda_i^{\perp}+\frac{\lambda_{xx}^{a1}-\lambda_{xx}^{a2}}{2(C_{11}-C_{12})},$$

where $a1$ and $a2$ mark such pockets lying near the two different reflection planes.

When the magnetic field is along the z axis, all $n_i(B)$ are one and the same for the equivalent Fermi surfaces, the contributions of the second terms in Λ_i^{a1} and Λ_i^{a1} to the sums over i cancel each other out, and we obtain from the above set of the equations that $u_{xx}=u_{yy}$, and

$u_{xy} = u_{zx} = u_{zy} = 0$. In this situation, the u_{zz} is the elongation $\Delta L/L$ of the crystal along the direction [001], whereas u_{xx} is the elongation in the direction [100]. Formulas (3) and (4) show that the constants Λ_i^c and $\Lambda_i^\perp$ extracted from the measurements of u_{zz} and u_{xx} enable one to find the constants $\lambda_{zz}^{(i)}$, $\bar\lambda_{xx}^{(i)}$ of the deformation potential if the the elastic moduli C_{mn} are known. In particular, these moduli for TaAs are given in Ref. [9], and for this semimetal, formulas (3) and (4) yield the following equations:

$$\Lambda_i^c \approx (-8.75\lambda_{zz}^{(i)} + 4.94\bar\lambda_{xx}^{(i)}) \times 10^{-25}\text{cm}^3, \qquad (5)$$
$$\Lambda_i^\perp \approx (2.48\lambda_{zz}^{(i)} - 4.67\bar\lambda_{xx}^{(i)}) \times 10^{-25}\text{cm}^3,$$

where $\lambda_{zz}^{(i)}$ and $\bar\lambda_{xx}^{(i)}$ are expressed in eV. Note that formula (2) was used in a number of papers [4–6]. (In Refs. [6], corrections to Eq. (2) were also taken into account when the chemical potential is close to the edge of a Landau subband.) For definiteness, we shall discuss only u_{zz} in the subsequent Supplementary Notes 2-7.

When the magnetic field deviates from the z axis, the densities $n_i(B)$ for different equivalent pockets generally do not coincides, and in this case, $u_{xx} \neq u_{yy}$, and the off-diagonal $u_{ik} \neq 0$. The latter means that the diagonalization of the tensor u_{kl} gives its principal axes that differ from the axes x, y, z, and hence, the deformation of the crystal in such a tilted magnetic field is a superposition of its elongations (contractions) along the three directions different from the crystallographic axes. Such elongations change the shape of the sample. For example, if the sample at $B = 0$ is a rectangular parallelepiped with the axes x, y, z, it becomes an oblique parallelepiped. When B deviates from the z axis in the direction of the x axis, the cross sections of this parallelepiped by the planes y =const. are parallelograms, the angle of which differs from $\pi/2$ by a small amount of the order of u_{xz}. If we define ΔL as the change in the distance between the parallel surfaces of the parallelepiped, the relative elongation $\Delta L/L$ is still given by u_{zz} (or u_{xx}). Another consequence of the tilt of B is that the different values of the factors Λ_i^{a1} and Λ_i^{a2} for the equivalents pockets manifest themselves in the expression for u_{xx}, whereas the factor Λ_i^c is still one and the same for these pockets. This fact may lead to essentially different dependences of u_{xx} and u_{zz} on the direction of B (Supplementary Note 8).

Finally, let us comment on the applicability of the rigid-band approximation, which has been used above. In general case, the shift $\Delta\varepsilon_i(\mathbf{p}) = \sum_{k,l}\lambda_{kl}^{(i)}(\mathbf{p})u_{kl}$ of the ith electron energy band $\varepsilon_i(\mathbf{p})$ under the deformation depends on $\mathbf{p}$ due to the $\mathbf{p}$ dependence of $\lambda_{kl}^{(i)}(\mathbf{p})$ [8]. The deformation potential $\lambda_{kl}^{(i)}(\mathbf{p})$ essentially changes on the scale of the order of the size of the Brillouin zone, and so one can consider $\lambda_{kl}^{(i)}$ as constant for small pockets of the Fermi surface. However, a caution is required when a band of the trivial electrons is separated from a valence band by a sufficiently small gap Δ. In this case, the effective electron masses of the band are of the order of Δ/V^2 where V is a typical interband matrix element of the velocity operator ($V \sim 10^5 \div 10^6$ m/s). If the small gap Δ substantially depends on a deformation, the rigid-band approximation can fail for a small pocket of this band since the effective masses, and hence the shape of the band, noticeably change with the deformation. Another situation occurs for the Weyl nodes. These nodes are topologically protected, a small deformation cannot create a gap in the spectrum, and so one may expect the applicability of the rigid-band approximation to the case of the Weyl pockets.

Supplementary Note 2: Magnetostriction produced by Weyl electrons in noncentrosymmetric crystals

As was shown in Sec. 3.3 of Ref. [10], for a *noncentrosymmetric* crystal, a contribution of a Fermi pocket of Weyl quasiparticles to any thermodynamic quantity (including the magnetostriction) can be calculated as half of the appropriate contribution of the Dirac pocket with the same electron dispersion. Using formulas for the electron spectrum in the magnetic field in the vicinity of a Dirac point [10, 11], the Ω potential and hence the density $n(B)$ of the Weyl electrons can be found. In particular, we obtain at zero temperature,

$$n(\zeta - \varepsilon_d, B) = n(\zeta - \varepsilon_d, 0)\Big(\frac{3}{4u} + \frac{3}{2u^{3/2}}\sum_{n=1}^{n=[u]}\sqrt{u-n}\Big), \qquad (6)$$

where $n(\zeta - \varepsilon_d, B)$ is the density of the Weyl quasiparticles in the magnetic field $H = B/\mu_0$ at a given chemical potential ζ; the density in absence of the magnetic field, $n(\zeta - \varepsilon_d, 0)$, is assumed to be positive in the case of the electrons ($\zeta - \varepsilon_d > 0$) and negative for the holes ($\zeta - \varepsilon_d < 0$); ε_d is the energy of the Weyl point; $[u]$ means the integer part of $u \equiv F/B$; the frequency of the quantum oscillations

$$F = \frac{S_{\max}}{2\pi e\hbar} \qquad (7)$$

also defines the boundary of the ultra-quantum region at which these oscillations disappear, and $S_{\max}$ is the maximal cross sectional area of the Fermi surface of the Weyl quasiparticles at a given direction of the magnetic field. This area is proportional to $(\zeta - \varepsilon_d)^2$. Of course, formula (6) is applicable to the case of the Dirac electrons, too. We also point out the two useful representations of the sum in Eq. (6),

$$\sum_{n=1}^{n=[u]}\sqrt{u-n} = \zeta(-1/2, \{u\}) - \zeta(-1/2, u), \qquad (8)$$

$$\sum_{n=1}^{n=[u]}\sqrt{u-n} = -\text{Im}\{\zeta(-1/2, 1-u-0i)\}, \qquad (9)$$

where $\zeta(-1/2, u)$ is the Hurwitz zeta function [12], and $\{u\} \equiv u - [u]$ is the fractional part of u.

We emphasize that formula (6) is valid for the most general form of the dispersion laws $\varepsilon_c(\mathbf{p})$ and $\varepsilon_v(\mathbf{p})$ describing the quasiparticles in the vicinity of the Weyl point [10],

$$\varepsilon_{c,v}(\mathbf{p}) = \varepsilon_d + \mathbf{a}\cdot\mathbf{p} \pm E(\mathbf{p}),$$
$$E(\mathbf{p}) = [b_{11}p_1^2 + b_{22}p_2^2 + b_{33}p_3^2]^{1/2},$$

where $\mathbf{a} = (a_1, a_2, a_3)$ and b_{ii} are the constant parameters, with a_i specifying the so-called tilt of the spectrum. For reference, let us also write the expressions for $n(\zeta - \varepsilon_d, 0)$ and $S_{\max}$ in terms of these parameters,

$$n(\zeta - \varepsilon_d, 0) = \frac{(\zeta - \varepsilon_d)^3}{3\pi^2\hbar^3(b_{11}b_{22}b_{33})^{1/2}(1-\tilde a^2)^2},$$
$$S_{\max} = \frac{\pi(\zeta - \varepsilon_d)^2}{(1-\tilde a^2)R_n^{1/2}},$$

where $\tilde a^2 \equiv \tilde a_1^2 + \tilde a_2^2 + \tilde a_3^2$, $\tilde a_i \equiv a_i/\sqrt{b_{ii}}$,

$$R_n = \sum_{i,j=1}^{3} \kappa^{ij} n_i n_j$$
$$\kappa^{ij} = \frac{b_{11}b_{22}b_{33}}{(b_{ii}b_{jj})^{1/2}}\left[(1-\tilde a^2)\delta_{ij} + \tilde a_i \tilde a_j\right],$$

δ_{ij} is the Kronecker symbol, and $\mathbf{n} = (n_1, n_2, n_3)$ is the unit vector along the magnetic field.

Formulas (2) and (6) describe the magnetostriction u_{zz} produced by the Weyl electrons at zero temperature T and in absence of the electron scattering by impurities. It was demonstrated by Shoenberg [13] that the effect of the impurities on the oscillating part of the magnetization can be taken into account by replacing the Fermi energy E_F (i.e. ζ at $T = 0$) by $E_F + i\Gamma$ where the parameter $\Gamma = \pi T_D$ characterizes the electron scattering by the impurities, and T_D is the so-call Dingle temperature. We shall use this replacement both for the oscillating and for the smooth parts of the magnetostriction. In other words, to take into account the scattering, the parameter $u + 0i$ in formula (9) is replaced by $u(1+i\gamma)^2$ where $\gamma \equiv \Gamma/(\zeta - \varepsilon_d) = \pi t_D$ is the dimensionless characteristic of the scattering, and t_D is the dimensionless Dingle temperature. As to the magnetostriction at $T > 0$, it can be obtained as follows:

$$u_{zz}(\zeta, T, B) = \int_{-\infty}^{\infty} d\varepsilon \frac{u_{zz}(\varepsilon, 0, B)}{4T\cosh^2[(\varepsilon - \zeta)/2T]}. \qquad (10)$$

Consider now various limiting cases.

Ultra-quantum regime ($\mu_0 H > F$). At high fields $\mu_0 H > F$, the sum in equation (6) disappears since $u = F/B < 1$ in this case. Then, with formula (2), we obtain the following contribution of a Weyl pocket to the magnetostriction:

$$u_{zz} = \Lambda n(\zeta - \varepsilon_d, 0)\left(\frac{3B}{4F} - 1\right) \equiv a + bB, \qquad (11)$$

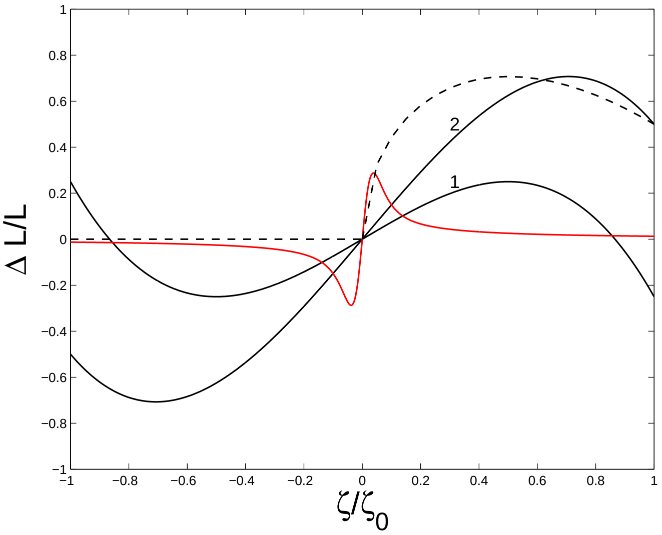

Supplementary Fig. 4. The magnetostriction $\Delta L/L = u_{zz}$ (in units of $\Lambda n(\zeta_0, 0)$) produced by the Weyl electrons at $T = 0$ versus the dimensionless chemical potential ζ/ζ_0 for $\mu_0 H/F = 2$ and $\mu_0 H/F = 1$ (the solid black lines, the numbers near these lines indicate the values of $\mu_0 H/F$). The chemical potential ζ is measured from the ε_d, and ζ_0 is its initial value. The solid red line shows the smooth part of the magnetostriction, $\bar u_{zz}$, enlarged in 10 times for $\mu_0 H/F = 1/7$ and for the nonzero temperature $T/\zeta_0 = 0.02$. The dashed line depicts the dependence of the magnetostriction on the chemical potential ζ at $T = 0$, $\mu_0 H/F = 2$ for the crystal with the electron spectrum (18) and $\delta = 1/2$ (the chemical potential is now measured from ε_0). Note that this dependence, which vanishes below ε_0, qualitatively differs from the dependence for the Weyl electrons.

where the quantities Λ, $n(\zeta - \varepsilon_d, 0)$, F introduced above and $a \equiv -\Lambda n(\zeta - \varepsilon_d, 0)$, $b \equiv -0.75a/F$ refer to this pocket. It is evident that the Weyl pocket produces the elongation $\Delta L/L$ that increases linearly with B at the high magnetic fields.

Consider the dependence of u_{zz} on the chemical potential ζ at a fix value of B (Supplementary Fig. 4). Since $F \propto S_{\max} \propto (\zeta - \varepsilon_d)^2$ and $n(\zeta - \varepsilon_d, 0) \propto (\zeta - \varepsilon_d)^3$, we arrive at

$$u_{zz} = \Lambda n(\zeta_0 - \varepsilon_d, 0)\frac{(\zeta - \varepsilon_d)^3}{(\zeta_0 - \varepsilon_d)^3}\left(\frac{3B}{4F}\frac{(\zeta_0 - \varepsilon_d)^2}{(\zeta - \varepsilon_d)^2} - 1\right),$$

where ζ_0 is the initial value of the chemical potential. Supplementary figure 4 shows that the magnetostriction changes its sign when ζ crosses the energy of the Weyl point ε_d.

Weak magnetic fields ($\mu_0 H \ll F$). At $\mu_0 H \ll F$, the ratio u in Eq. (6) is large, and the sum contains many terms. Using representation (8) and the expansion of the Hurwitz zeta function $\zeta(-1/2, u)$ at $u \gg 1$ [12],

$$\zeta(-1/2, u) \approx -\frac{2}{3}u^{3/2} + \frac{1}{2}u^{1/2} - \frac{1}{24u^{1/2}}, \qquad (12)$$

we find from formulas (2) and (6) that the magnetostriction u_{zz} produced by a Weyl pocket comprises the smooth and oscillating parts in the weak-field range, $u_{zz} = \bar{u}_{zz} + u_{zz}^{\rm osc}$. The smooth part $\bar{u}_{zz}$ has the form

$$\bar{u}_{zz} = \Lambda n(\zeta - \varepsilon_d, 0)\frac{B^2}{16F^2} = -\frac{b^2}{9a}B^2 \equiv cB^2, \qquad (13)$$

and the oscillating part $u_{zz}^{\rm osc}$ results from the term $\zeta(-1/2, \{u\})$ in Eq. (8),

$$u_{zz}^{\rm osc} = \Lambda n(\zeta - \varepsilon_d, 0)\frac{3\zeta(-1/2, \{u\})}{2u^{3/2}}. \qquad (14)$$

The oscillating behavior of $u_{zz}^{\rm osc}$ with changing $u = F/B$ becomes evident from the formula [12],

$$\zeta(-1/2, \{u\}) = \frac{1}{2\pi\sqrt{2}}\sum_{n=1}^{\infty}\frac{1}{n^{3/2}}\sin\!\Big(2\pi n u - \frac{\pi}{4}\Big). \qquad (15)$$

Interestingly, with changing the chemical potential, the coefficient c sharply changes it sign when ζ crosses the energy of the Weyl point (Supplementary Fig. 4),

$$c(\zeta) = c(\zeta_0)\frac{(\zeta_0 - \varepsilon_d)}{(\zeta - \varepsilon_d)}, \qquad (16)$$

where ζ_0 is the initial value of ζ. Note that $c(\zeta)$ diverges at $\zeta = \varepsilon_d$.

Formulas (11), (13), (14), and (16) are written at $T = 0$. A nonzero temperature tends to suppress the oscillations of the magnetostriction, but it has effect on $\bar{u}_{zz}$ only in the region $|\zeta - \varepsilon_d| \lesssim T$. In this region, the divergence of $c(\zeta)$ near ε_d is cut off (Supplementary Fig. 4), and the maximal value of $c(\zeta)$ is of the order of $c(\zeta_0)(\zeta_0 - \varepsilon_d)/T$.

Finally, as an example, we calculate the magnetostriction produced by the two groups of the Weyl electrons (W1 and W2) and by the holes, assuming that the contribution of these holes to the magnetostriction are described by the term $c_h B^2$ where c_h is a constant. Let experimental data on the magnetostriction in the ultra-quantum regime for the W1 and W2 electrons can be approximated by the polynomial $a + bB + c_h B^2$. Then, if the frequencies F_{W1}, F_{W2} are known, the constants a_{W1} and a_{W2} are found from the equations that follows from formula (11),

$$a_{W1} + a_{W2} = a,$$
$$-0.75\left(\frac{a_{W1}}{F_{W1}} + \frac{a_{W2}}{F_{W2}}\right) = b.$$

With known a_{W1}, a_{W2}, F_{W1}, F_{W2}, the magnetostriction at an arbitrary B is calculated, using formulas (2) and (6). The dimensionless Dingle temperatures are determined from the best fit of the calculated curve at $T = 0$ to the appropriate experimental data at a low temperature. In particular, we find for TaAs that $F_{W1} = 7.2$ T, $a = -2.18 \times 10^{-6}$, $b = 2.55 \times 10^{-7}$ T^{-1}, $c_h = 1.49 \times 10^{-8}$ T^{-2}. In the case of $F_{W2} = 5$ T, the calculated magnetostriction together with the experimental data are presented in Supplementary Fig. 5.

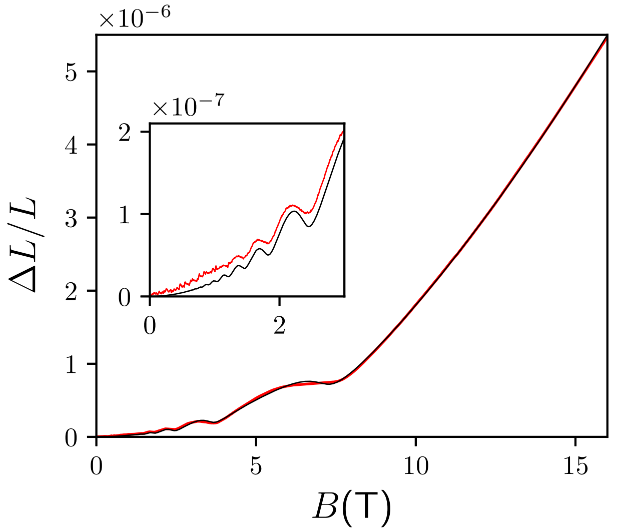

Supplementary Fig. 5. Comparison of the calculated magnetostriction with the experimental data for TaAs. The red line shows the c-axis magnetostriction $\Delta L/L$ of the crystal TaAs measured at $T = 25$ mK for the magnetic fields aligned with this axis (see Fig. 2a in the main text). The black line depicts the magnetostriction calculated at zero temperature, using equations (2), (6) and (9) for the W1 and W2 electrons and assuming that $c_h B^2$ is the contribution of the holes to the magnetostriction. We use the following values of the parameters: $c_h = 1.49 \times 10^{-8}$ T^{-2}, $a_{W1} = -1.58 \times 10^{-6}$, $a_{W2} = -0.6 \times 10^{-6}$, $F_{W1} = 7.2$ T, $F_{W2} = 5$ T, $\gamma_{W1} = 0.025$ and $\gamma_{W2} = 0.1$.

Supplementary Note 3: Magnetostriction produced by electrons with parabolic spectrum

For comparison, consider the magnetostriction u_{zz} produced by the "trivial" electrons with the parabolic dispersion,

$$\varepsilon(p_x, p_y, p_z) = \varepsilon_0 + \frac{p_x^2}{2m_x} + \frac{p_y^2}{2m_y} + \frac{p_z^2}{2m_z}, \qquad (17)$$

where ε_0 is the edge of the electron band, and m_x, m_y, m_z are the effective electron masses. In the magnetic field H these electrons have the well-known energy spectrum,

$$\varepsilon_n(p_\parallel) = \varepsilon_0 + \frac{eB\hbar}{m_*}(n + \frac{1}{2} + \delta) + \frac{p_\parallel^2}{2m_\parallel}, \qquad (18)$$

where n is an integer ($n \geq 0$); $p_\parallel$ is the quasimomentum along the magnetic field; $m_\parallel$ and m_* are the longitudinal effective mass and the cyclotron mass, respectively, and the electron magnetic moment composed of its spin and orbital parts is written as $\delta(e\hbar/m_*)$, with δ being a constant. Since due to the time reversal symmetry, the contribution of any charge-carrier pocket to the Ω potential for a noncetrosymmetric crystal can be considered as a

half contribution of the same pocket with the states that are doubly degenerate in spin, one should imply that the parameter δ in the Zeeman term $\delta(e\hbar B/m_*)$ of Eq. (18) takes the values $+\delta$ and $-\delta$ (in this situation, δ can be represented in terms of the g factor, $\delta = gm_*/4m$). With this spectrum, we obtain at zero temperature,

$$n(\zeta-\varepsilon_0,B)=\frac{3n(\zeta-\varepsilon_0,0)}{4u^{3/2}}\sum_{\pm,n=0}^{n=[u-0.5\mp\delta]}\sqrt{u-\frac{1}{2}\mp\delta-n}, \quad (19)$$

where $n(\zeta-\varepsilon_0,B)$ is the density of the quasiparticles in the magnetic field $H = B/\mu_0$ at a given chemical potential ζ; the frequency F in the ratio $u \equiv F/B$ is still defined by formula (7). However, for the trivial electrons, the maximal cross sectional area $S_{\max}$ of their Fermi surface is proportional to $(\zeta-\varepsilon_0)$.

Ultra-quantum regime. At $\delta < 1/2$ and $\mu_0H > B_{uq} = F/(0.5-\delta)$, the sum in equation (19) disappears. Then, with formula (2), we obtain the constant contribution of the trivial-electron pocket to the magnetostriction in this ultra-quantum limit,

$$u_{zz} = -\Lambda n(\zeta-\varepsilon_0,0) \equiv a. \quad (20)$$

Interestingly, at $\delta = 1/2$, this limiting value of u_{zz} cannot be reached since at any H, the lowest Landau subband remains occupied by the electrons. In this situation Eq. (19) reduces to formula (6) for the Weyl electrons.

When $\delta > 1/2$, the behavior of the magnetostriction in the ultra-quantum limit cardinally changes. It follows from Eq. (19) that

$$n(\zeta-\varepsilon_0,B)=\frac{3n(\zeta-\varepsilon_0,0)}{4u^{3/2}}\sqrt{u-\frac{1}{2}+\delta}, \quad (21)$$

$$n(\zeta-\varepsilon_0,B)=\frac{3n(\zeta-\varepsilon_0,0)}{4u^{3/2}}\left[\sqrt{u-\frac{1}{2}+\delta}+\sqrt{u-\frac{3}{2}+\delta}\right],$$

for $1/2 < \delta < 3/2$ at $\mu_0H > F/(1.5-\delta)$ and for $3/2 < \delta < 5/2$ at $\mu_0H > F/(2.5-\delta)$, respectively. In both the cases, $n(\zeta-\varepsilon_0,B) \propto B^{3/2}$ at $B \gg F$. However, if a limited range of the high magnetic fields is considered, formulas (21) lead to the curves $u_{zz}(B)$, the shapes of which are close to the straight line described by Eq. (11) (cf. Fig. 1 in the main text). Nevertheless, the slopes of these curves $\lambda \equiv du_{zz}/dB$ depend on δ and differ from the slope characteristic of the Weyl quasiparticles. Indeed, the slope for the Weyl fermions, $\lambda = b = 0.75\Lambda n(\zeta-\varepsilon_d,0)/F$, is determined by the frequency F only if u_{zz} is measured in units of $\Lambda n(\zeta-\varepsilon_d,0) = -a$.

The special case $\delta = 1/2$, for which the formula for the magnetostriction exactly coincides with the expression for the Weyl electron, separates the two essentially different behaviors of the magnetostriction in the ultra-quantum limit.

Weak magnetic fields ($\mu_0H \ll F$). At $\mu_0H \ll F$, the ratio u in Eq. (19) is large, and the sum contains many terms. Similarly to the case of the Weyl electrons, we find from formulas (2) and (19) that the magnetostriction u_{zz} produced by the trivial electrons comprises the smooth and oscillating parts in the weak-field range, $u_{zz} = \bar{u}_{zz} + u_{zz}^{\rm osc}$. These smooth ($\bar{u}_{zz}$) and oscillating ($u_{zz}^{\rm osc}$) parts have the following forms:

$$\bar{u}_{zz} = \Lambda n(\zeta-\varepsilon_0,0)\frac{3B^2}{8F^2}(\delta^2-\frac{1}{12}) \equiv cB^2, \quad (22)$$

$$u_{zz}^{\rm osc} = \frac{3\Lambda n(\zeta-\varepsilon_0,0)}{4u^{3/2}}\sum_{\pm}\zeta(-1/2,\{u-0.5\pm\delta\}), \quad (23)$$

where $\{x\} \equiv x - [x]$ is fractional part of x. Interestingly, at $\delta \to 0$ when the Zeeman energy is negligible as compared to the cyclotron spacing between the Landau subbands, we find that $c = -\Lambda n(\zeta-\varepsilon_0,0)/(32F^2)$. This value is of the opposite sign as compared to c for the Weyl electrons. On the other hand, the phase of the oscillating part (23) at $\delta \to 0$ is shifted by π with respect to the phase of the oscillations described by Eq. (14). This shift is caused by the difference in the Berry phases for these two cases [10, 14]. On the other hand, at $\delta = 1/2$ the constant c exactly coincides with that given by Eq. (13), and equation (23) for the oscillating part reduces to Eq. (14).

Thus, we find that the B-dependence of u_{zz} for the trivial electrons with $\delta \neq 1/2$ essentially differs from the appropriate dependence for the Weyl quasiparticles (see also Fig. 1 in the main text). This result enables one to distinguish between the Weyl and trivial electrons with measurements of the magnetic-field dependences of the magnetostriction. However, if $\delta = 1/2$, the formulas for the magnetostriction exactly coincide in these two cases.

According to Ref. [15, 16], the quantity δ is close to 1/2 for a band in a *centrosymmetric* crystal if this band is separated from another by a small gap that is less than the strength of the spin-orbit interaction. These bands are doubly degenerate in spin for such crystals. Strictly speaking, the spectrum (18) (with $\pm\delta$) is applicable to this situation only if the chemical potential measured from the edge of the band, $\zeta-\varepsilon_0$, is noticeably less than the gap Δ in the spectrum. However, it turns out that even if $\zeta-\varepsilon_0 \gtrsim \Delta$, the B dependence of the magnetostriction for the gapped spectrum coincides with its B dependence for Dirac electrons. In this context, it is worth noting that the spectrum of bismuth is sufficiently well described by a two-band model, the parameter δ is close to 1/2 [16], and so the field dependence of its magnetostriction [5, 17] is reminiscent of the appropriate dependence for the Dirac electrons. However, the situation essentially changes for the *noncentrosymmetric* Weyl semimetals considered here. In particular, the holes in TaAs are located near the nodal ring that would occur in this semimetal in neglect of the spin-orbit interaction [18]. The spin-orbit coupling lifts the degeneracy of the bands, and there are *four* nearby bands in the region of the Brillouin zone where the holes exist. In this situation, there is no reason to expect that $\delta \approx 1/2$ for them.

As is clear from Supplementary Note 1, if not only ε_0 but also the gap in the spectrum Δ depend on a deformation (and $\zeta - \varepsilon_0$ is not-too-small as compared to this gap), the rigid-band approximation can fail. In this case, formula (2) will contain an additional term, and in the weak-field range, this term is proportional to B^2.

Supplementary Note 4: Magnetostriction and magnetization

Let us compare the magnetostriction with the magnetization, which is considered as another thermodynamic probe of the Weyl electrons [19–21]. The magnetization M characterizes the change of the electron energy in the magnetic field, and this change occurs for all electrons, including those lying far below the Fermi level. In particular, a band completely filled by electrons can give a noticeable contribution $\chi_0 H$ to the magnetization where χ_0 is a constant. It is significant that the absolute value of this constant increases when a top of the filled band approaches the lowest boundary of unoccupied electron states at the same point of the Brillouin zone. This increase is due to virtual interband transitions of electrons in the magnetic field. On the other hand, the magnetostriction probes the energy of the interaction between electrons and the elastic deformations of the crystal, and within the rigid-band approximation, it is determined by the change of the charge-carrier density in the magnetic field. This density remains unchanged for the filled band, and so this band does not contribute to the magnetostriction.

In the weak magnetic fields, the magnetization of trivial electrons is proportional to $(\zeta - \varepsilon_0)^{1/2} B \propto n^{1/3} B$ [8] and increases with increasing n. In contrast, the coefficient c in Eq. (22), which characterizes the change of the electron density in the magnetic field, decreases with n,

$$c \propto \frac{n}{F^2} \propto (\zeta - \varepsilon)^{-1/2} \propto n^{-1/3}.$$

As to a Weyl point, it was found (see review [10] and the reference therein) that the appropriate magnetization looks like $M \propto \ln(|E_F - \varepsilon_d|) B \propto \ln(n^{1/3}) B$. In other words, the smaller Weyl pocket, the larger contribution of the Weyl point to the M, and this increase is reminiscent of that given by Eq. (16) for the coefficient c:

$$c \propto (\zeta - \varepsilon_d)^{-1} \propto n^{-1/3}.$$

However, these enhancements of the magnetization and of the magnetostriction have different origin. The magnetostriction of the Weyl electrons grows to higher values due to the decrease of their pocket, whereas in the case of the magnetization, its logarithmic enhancement is caused by the lower filled band since the Fermi level, i.e., the boundary of the unoccupied electron states, tends to the top of this band ε_d when the Weyl pocket shrinks.

In the ultra-quantum limit, the magnetization of the Weyl electrons is proportional to $B \ln(CB/F) - 6F$ where C is a constant [10, 11, 22], see also [19, 20]. Except for the logarithmic factor, this formula looks like Eq. (11). However, this factor has the same origin as the factor $\ln|E_F - \varepsilon_d|$ in the weak-field expression, and it can be obtained by the replacement of the difference $|E_F - \varepsilon_d|$ by the Landau-level spacing, which becomes larger than this difference in the ultra-quantum limit. As to the magnetization of the trivial electrons in this limit, it tends to zero [20] if the parameter $\delta < 1/2$ and is proportional to $B^{3/2}$ in the opposite case of $\delta > 1/2$. (Indeed, for $\delta > 1/2$, simple estimates give: $n \propto B^{3/2}$, the B-dependent contribution to the Ω potential, $\delta\Omega \propto (\delta - 1/2)(e\hbar B/m_*)n$, and $M = -\partial\delta\Omega/\partial B \propto B^{3/2}$.) By and large we may conclude that although the results for the magnetostriction and the magnetization are quite similar, they stem from the different contributions to the thermodynamic potential and have different physical origin.

Supplementary Note 5: Magnetostriction of TaAs

In Supplementary Notes 2 and 3 the B-dependences of the magnetostriction are analyzed under the assumption that a variation of the magnetic field does not change the chemical potential ζ of electrons. This situation does can occur when a crystal contains a large charge-carrier group that maintains the constancy of the chemical potential. However, in TaAs there are eight equivalent pockets of the W1 electrons, sixteen equivalent pockets of the W2 electrons, and eight equivalent pockets of the holes, with all the pockets being relatively small. In this case it is necessary to take into account the magnetic-field dependence of the chemical potential in analyzing the magnetostriction. This dependence $\zeta(B)$ is found from the conservation condition of the total density of the charge carriers,

$$\sum_i [n_i(\zeta, B) - n_i(\zeta_0, 0)] = 0, \qquad (24)$$

where the summation is over all the pockets, ζ_0 is the initial value of ζ at $H = 0$, and the charge-carrier density in the magnetic field for the ith pocket, $n_i(\zeta, B)$, is described by formula (6) for the Weyl electrons and by Eq. (19) for the holes [or by some other expression if the spectrum of the holes differs from that given by Eq. (18)]. With the B-dependence of ζ, formula (2) is rewritten as follows:

$$u_{zz} = \sum_i^N \Lambda_i [n_i(\zeta, B) - n_i(\zeta_0, 0)]. \qquad (25)$$

It is clear from Eqs. (24) and (25) that if all the constants Λ_i were equal, the magnetostriction u_{zz} would be zero.

Consider a special case when the magnetic field is considerably less than the fields F_i/μ_0 for all the groups of

the charge carriers. In this situation, equation (24) in the chemical potential ζ is approximately solvable since the difference $n_i(\zeta,B)-n_i(\zeta_0,0)$ can be rewritten as follows:

$$n_i(\zeta,B)-n_i(\zeta_0,0)=n_i(\zeta,B)-n_i(\zeta_0,B)\\ +n_i(\zeta_0,B)-n_i(\zeta_0,0)\approx\nu_i(\zeta-\zeta_0)\\ +n_i(\zeta_0,B)-n_i(\zeta_0,0),$$

where $\nu_i\equiv\partial n_i(\zeta_0,0)/\partial\zeta_0$, and we have taken into account that $\partial n_i(\zeta,B)/\partial\zeta\approx\nu_i$ at the weak magnetic fields $\mu_0H\ll F_i$. Then, equations (24) and (25) give

$$(\zeta-\zeta_0)=\frac{\sum_i[n_i(\zeta_0,B)-n_i(\zeta_0,0)]}{\nu},\\ u_{zz}=\sum_i\tilde\Lambda_i[n_i(\zeta_0,B)-n_i(\zeta_0,0)], \qquad (26)\\ \tilde\Lambda_i=\sum_m\frac{\nu_m}{\nu}(\Lambda_i-\Lambda_m),$$

where $\nu\equiv\sum_i\nu_i$. Therefore, in this weak-field range the B-dependence of the chemical potential can be taken into account by the renormalization of the constants Λ_i. For stronger magnetic fields, it is necessary to solve equation (24) numerically.

In the case of TaAs, it is convenient to rewrite general formula (25) with the use of Eq. (24) as follows:

$$u_{zz}=(\Lambda_{W1}-\Lambda_h)\sum_{i=1}^{8}[n_i(\zeta,B)-n_i(\zeta_0,0)]\\ +(\Lambda_{W2}-\Lambda_h)\sum_{i=1}^{16}[n_i(\zeta,B)-n_i(\zeta_0,0)], \qquad (27)$$

where the summation is carried out over the W1 and W2 pockets only; the constants Λ_{W1}, Λ_{W2}, and Λ_h refer to the W1, W2 electrons, and to the holes, respectively. This formula is valid at any strength of the magnetic fields. In the weak magnetic fields, $\mu_0H\ll F_i$, formulas (24) and (27) are equivalent to Eqs. (26).

Magnetic field parallel to the c axis. When the magnetic field is parallel to the c axis, all the pockets in the W1 electron group or in the W2 group or in the hole group give identical contributions to the magnetostriction. Let F_{W1}, F_{W2}, and F_h denote the frequencies F_i for the W1 and W2 electrons pockets and for the holes, respectively. According to Ref. [18], at this direction of B, one has $F_{W1}\sim7$ T, $F_{W2}\sim5$ T, and the field $F_h\sim19$ T for the holes is larger than the maximal field 16 T in our experiments. Since F_h is large, we shall consider the range $\mu_0H\le16$ T as the low-field region for the holes and shall describe their contribution to Eq. (24) as follows:

$$n_h(\zeta,B)-n_h(\zeta_0,0)=n_h(\zeta,B)-n_h(\zeta,0)\\ +n_h(\zeta,0)-n_h(\zeta_0,0)\\ \approx B^2\Big(\beta(\zeta_0)+\frac{d\beta(\zeta_0)}{d\zeta_0}(\zeta-\zeta_0)\Big)+\nu_h(\zeta_0)(\zeta-\zeta_0), \qquad (28)$$

where $\nu_h=\partial n_h(\zeta,0)/\partial\zeta$ is density of states for the holes in zero magnetic field, whereas the function $\beta(\zeta)$ defines the variation of the hole density for the low magnetic fields,

$$n_h(\zeta,B)-n_h(\zeta,0)=\beta(\zeta)B^2.$$

(The oscillation contribution to $n_h(\zeta,B)$ is neglected.) If the holes can be described by the parabolic spectrum (17), (18), one arrives at [compare with Eq. (22)],

$$\beta(\zeta)=\frac{3n_h(\zeta,0)}{8F_h^2}(\delta^2-\frac{1}{12}),\\ \frac{d\beta(\zeta)}{d\zeta}=\frac{\beta(\zeta)}{2(\varepsilon_0-\zeta)}, \qquad (29)\\ \nu_h(\zeta)=-\frac{3n_h(\zeta,0)}{2(\varepsilon_0-\zeta)},$$

where ε_0 is the edge of the hole band, and we have taken into account that $n_h(\zeta,0)\propto-(\varepsilon_0-\zeta)^{3/2}$ and $n_h(\zeta,0)/F_h^2(\zeta)\propto-(\varepsilon_0-\zeta)^{-1/2}$ for the parabolic spectrum. However, we emphasize that formula (28) is applicable to the holes even though their spectrum is not described by the parabolic model, and their Fermi surface noticeably differs from an ellipsoid. The only requirement of the applicability is the condition of the low-field limit for them (i.e., $B<F_h$).

Let us introduce the dimensionless deviation of the chemical potential from its initial value at $H=0$, $\delta\tilde\zeta\equiv(\zeta-\zeta_0)/(\zeta_0-\varepsilon_{d,W1})$ where $\varepsilon_{d,W1}$ is the energy of the Weyl points W1. Then, at $T=0$, equation (24) that determines $\delta\tilde\zeta(B)$ is rewritten as follows:

$$\left(1-\frac{3(1+\delta\tilde\zeta)}{4u_1}-\frac{3}{2u_1^{3/2}}\sum_{n=1}^{n=[u_1]}\sqrt{u_1(1+\delta\tilde\zeta)^2-n}\right)\\ +\frac{n_{W2}}{n_{W1}}\left(1-\frac{3(1+v\delta\tilde\zeta)}{4u_2}-\frac{3}{2u_2^{3/2}}\sum_{n=1}^{n=[u_2]}\sqrt{u_2(1+v\delta\tilde\zeta)^2-n}\right)\\ +B^2(\tilde\beta_0+\tilde\beta_1\delta\tilde\zeta)+\tilde\nu_h\delta\tilde\zeta=0, \qquad (30)$$

where n_{W1}, n_{W2} are the densities of the electrons at zero magnetic field (i.e., at $\zeta=\zeta_0$); $v\equiv(\zeta_0-\varepsilon_{d,W1})/(\zeta_0-\varepsilon_{d,W2})$; $\varepsilon_{d,W2}$ is the energy of the Weyl points W2,

$$\tilde\beta_0\equiv-\frac{\beta(\zeta_0)}{n_{W1}},\\ \tilde\beta_1\equiv-\frac{(\zeta_0-\varepsilon_{d,W1})}{n_{W1}}\frac{d\beta(\zeta_0)}{d\zeta_0},\\ \tilde\nu_h\equiv-\frac{(\zeta_0-\varepsilon_{d,W1})}{n_{W1}}\nu_h(\zeta_0);$$

$u_1=F_{W1}/B$, $u_2=F_{W2}/B$, and the frequencies F_{W1}, F_{W2} are taken at $\zeta=\zeta_0$. The densities n_i satisfies the relation $n_{W1}+n_{W2}-|n_h|=n_{\rm imp}$ where n_h is the hole density at $H=0$, and the charge carrier density $n_{\rm imp}$

is caused by impurities. This $n_{\rm imp}$ specifies the doping in the sample. With the doping, $1+(n_{W2}/n_{W1})-(|n_h|/n_{W1})\neq 0$.

In the case of the parabolic spectrum for the holes, we obtain from Eqs. (29),

$$\tilde\beta_0=\frac{3|n_h|}{(8F_h^2 n_{W1})}(\delta^2-\frac{1}{12}),$$
$$\frac{\tilde\beta_1}{\tilde\beta_0}=\frac{(\zeta_0-\varepsilon_{d,W1})}{2(\varepsilon_0-\zeta_0)}, \qquad (31)$$
$$\tilde\nu_h=-\frac{3|n_h|(\zeta_0-\varepsilon_{d,W1})}{2n_{W1}(\varepsilon_0-\zeta_0)}=-\frac{3|n_h|}{n_{W1}}\frac{\tilde\beta_1}{\tilde\beta_0}.$$

Interestingly, if the magnetic field is tilted away from the c axis, the quantities F_h, δ, and hence $\tilde\beta_0$, will change, but the ratio $\tilde\beta_1/\tilde\beta_0$ will remain unchanged for the parabolic spectrum. Of course, the parameters $\tilde\nu_h$, $\tilde\beta_0$, and $\tilde\beta_1$ may essentially differ from these estimates if the dispersion of the holes noticeably deviates from the parabolic law.

According to expression (27), the general formula for the magnetostriction $\Delta L/L=u_{zz}$ of TaAs at zero temperature takes the form:

$$\frac{\Delta L}{L}=A_{W1}\Big(1-\frac{3(1+\delta\tilde\zeta)}{4u_1}-\frac{3}{2u_1^{3/2}}\sum_{n=1}^{n=[u_1]}\sqrt{u_1(1+\delta\tilde\zeta)^2-n}\Big)+A_{W2}\Big(1-\frac{3(1+v\delta\tilde\zeta)}{4u_2}-\frac{3}{2u_2^{3/2}}\sum_{n=1}^{n=[u_2]}\sqrt{u_2(1+v\delta\tilde\zeta)^2-n}\Big), \qquad (32)$$

where $A_{W1}\equiv-(\Lambda_{W1}-\Lambda_h)n_{W1}$, $A_{W2}\equiv-(\Lambda_{W2}-\Lambda_h)n_{W2}$, and $\delta\tilde\zeta(H)$ is found from Eq. (30).

To take into account the electron scattering by impurities, we use the representation (9) for the sums in Eqs. (30) and (32) and replace ζ by $\zeta+i\Gamma_i$ where the quantity Γ_i characterizes the scattering of charge carriers in the pocket i (i =W1, W2); see the Supplementary Note 2. This means that $\delta\tilde\zeta$ is replaced by $\delta\tilde\zeta+i\gamma_{W1}$ or $\delta\tilde\zeta+i(\gamma_{W2}/v)$ in the appropriate terms of Eqs. (30) and (32) where $\gamma_i=\Gamma_i/(\zeta-\varepsilon_{Wi})$.

The magnetostriction at nonzero temperature can be calculated with formula (10). In order to apply this formula, let us introduce the new variable $x\equiv(\varepsilon-\zeta)/(\zeta_0-\varepsilon_{d,W1})$ in integral (10) and the dimensionless temperature $t\equiv T/(\zeta_0-\varepsilon_{d,W1})$. Then, Eq. (10) is rearranged as follows:

$$u_{zz}(\zeta,T)=\frac{1}{4t}\int_{-\infty}^{\infty}dx\frac{u_{zz}(x,0)}{\cosh^2(x/2t)}, \qquad (33)$$

where $u_{zz}(x,0)$ is given by formula (32) in which the factors $(1+\delta\tilde\zeta)$ and $(1+v\delta\tilde\zeta)$ are replaced by $(1+\delta\tilde\zeta+x)$ and $(1+v\delta\tilde\zeta+vx)$, respectively.

Supplementary Note 6: Estimates of the parameters characterizing Fermi-surface pockets in TaAs

Parameters characterizing Weyl Fermi pockets can be estimated, using the data for the quantum-oscillation frequencies and for the appropriate cyclotron masses [23]. In particular, if the frequency F produced by a Weyl pocket and the appropriate cyclotron mass m_* have been measured at least for one direction of the magnetic field, the formula

$$|\zeta-\varepsilon_d|=\frac{S_{\rm max}}{\pi|m_*|}=\frac{2e\hbar F}{|m_*|}, \qquad (34)$$

enables one to find the position of the chemical potential ζ relative to the energy ε_d of the Weyl point. Here $S_{\rm max}$ is the area of the extremal cross section perpendicular to the magnetic field for the Weyl pocket. The density n_W of the Weyl charge carriers can be expressed in terms of directly-measurable frequencies of the quantum oscillations,

$$n_W=\frac{N_W V}{(2\pi\hbar)^3}, \qquad (35)$$

where V is the volume of a Weyl pocket in the Brillouin zone,

$$V=\frac{4[S_{\rm max}^{(1)}S_{\rm max}^{(2)}S_{\rm max}^{(3)}]^{1/2}}{3\pi^{1/2}}=\frac{8\sqrt{2}\pi(e\hbar)^{3/2}(F_1F_2F_3)^{1/2}}{3}, \qquad (36)$$

N_W is the numbers of the equivalent Weyl pockets, and F_i are the frequencies associated with the principal directions of the Fermi-surface ellipsoid. In other words, F_1 and F_3 are the maximal and minimal frequencies produced by the pocket when the magnetic field rotates in various planes, and F_2 corresponds to the direction of H perpendicular to the directions at which F_1 and F_2 occur. The cross sectional areas $S_{\rm max}^{(i)}$ correspond to the frequencies F_i, and these cross sections are mutually orthogonal.

In the case of the parabolic dispersion of charge carriers, the formula that is similar to Eq. (34) looks as follows:

$$|\zeta-\varepsilon_d|=\frac{S_{\rm max}}{2\pi|m_*|}=\frac{e\hbar F}{|m_*|}, \qquad (37)$$

and contains the additional factor 1/2 as compared to Eq. (34).

Using the experimental data for the cross-sectional areas and the cyclotron masses (Table I in Ref. [18]) and the calculated value $F_{W2}\sim 5$ T for H parallel to the c axis [18], we obtain with formulas (34)-(36) that $n_{W1}\approx 2.5\times10^{18}$ cm^{-3}, $\zeta_0-\varepsilon_{d,W1}\approx 28.4\pm3.5$ meV [23], and $n_{W2}/n_{W1}\sim 0.15$, $\zeta_0-\varepsilon_{d,W2}\approx 11.9\pm1$ meV. Since the oscillations in the magnetostriction of our sample and the quantum oscillations observed by Arnold et al. [18] for the W1 electrons have practically the same frequency

at H parallel to the c axis, we conclude that the Fermi-surface parameters estimated above are appropriate for our samples, too. For this reason, in the calculation with formulas of Supplementary Note 5, we set

$$v \equiv \frac{(\zeta_0 - \varepsilon_{d,W1})}{(\zeta_0 - \varepsilon_{d,W2})} = 2.5, \quad \frac{n_{W2}}{n_{W1}} = 0.15. \qquad (38)$$

Interestingly, in the case of the parabolic spectrum for the holes, we similarly can find $\varepsilon_0 - \zeta_0$ using Eq. (37) and values of F and m_* measured for the hole at two directions of H [18]. It turns that $\varepsilon_0 - \zeta_0$ lies in the interval 12.6–20.6 meV if the data for $H \parallel [100]$ are used, and $\varepsilon_0 - \zeta_0$ is in the range 7.9–16.2 meV if we use the data for $H \parallel [110]$. Therefore, it is reasonable to assume that 12.6 meV $< \varepsilon_0 - \zeta_0 <$ 16.2 meV. With this $\varepsilon_0 - \zeta_0$ and the above $\zeta_0 - \varepsilon_{d,W1}$, we arrive at the estimate,

$$\frac{\tilde\beta_1}{\tilde\beta_0} = \frac{(\zeta_0 - \varepsilon_{d,W1})}{2(\varepsilon_0 - \zeta_0)} \approx 0.8 \div 1.3.$$

If the dispersion of the holes deviates from the parabolic dependence, the estimate of $\varepsilon_0 - \zeta_0$ and the expression for $\tilde\beta_1/\tilde\beta_0$ can noticeably change. Below we do not exclude the possibility of this deviation since the hole pockets of the Fermi surface in TaAs are associated with the band-contact lines that would occur in absence of the spin-orbit interaction [18].

Supplementary Note 7: Calculation of the magnetostriction along the [001] direction and an analysis of the obtained parameters

Calculations. We calculate the magnetostriction with Eqs. (30), (32) at given frequencies $F_{W1} = 7.2$ T, $F_{W2} = 5$, and at the fixed values of the ratios $(\zeta_0 - \varepsilon_{d,W1})/(\zeta_0 - \varepsilon_{d,W2})$ and n_{W2}/n_{W1}, see Eq. (38). The values of the other parameters are chosen so that the calculated magnetostriction best matches the experimental data (set 1 in Supplementary Table I). The result is presented in Supplementary Fig. 6. Note that although the value $F_{W2} = 5$ T was calculated in Ref. [18], the frequency F_{W2} was not measured anywhere, and so it can, in principle, differ from this value. We find that if F_{W2} decreases, the quality of such fits of the theoretical curve to the experimental data gradually improves, and the best fit is reached at $F_{W2} \approx 1.35$ T; see set 2 in Supplementary Table I and Supplementary Fig. 7. In this context, we shall analyze the two sets of the parameters below. The first set agrees with the value of F_{W2} from Ref. [18], while the second one provides the best fit of the theoretical curve to the experimental data on the magnetostriction.

We also compare the magnetostriction measured at the temperature $T = 4.2$ K with the magnetostriction calculated at a finite dimensionless temperature $t = T/(\zeta_0 - \varepsilon_{W1})$, taking into account the dependence $\zeta(B)$. In Supplementary Fig. 8, we demonstrate this comparison in the case of the first set of the parameters in Supplementary Table I. The theoretical curve agrees with the experimental data at $t \approx 0.015$. (The same $t \approx 0.015$ is also obtained for the second set.) This value of t leads to the independent estimate of $\zeta_0 - \varepsilon_{W1}$: $\zeta_0 - \varepsilon_{W1} \approx 280$ K ≈ 24 meV, which is only a little less than the value 28.4 ± 3.5 meV obtained from the data of Arnold et al. [18] in Supplementary Note 6.

We may now estimate the Dingle temperature $T_{D,W1} = (\zeta_0 - \varepsilon_{W1})\gamma_{W1}/\pi$ for the W1 electrons. The data of Supplementary Table I and $\zeta_0 - \varepsilon_{W1} \approx 280$ K give $T_{D,W1} \approx 2.2$ K. This value of $T_{D,W1}$ is comparable with $T_{D,W1} \approx 3.2$ K obtained in Ref. [18]. As to $T_{D,W2}$, we can only tentatively estimate its value $T_{D,W2} =$

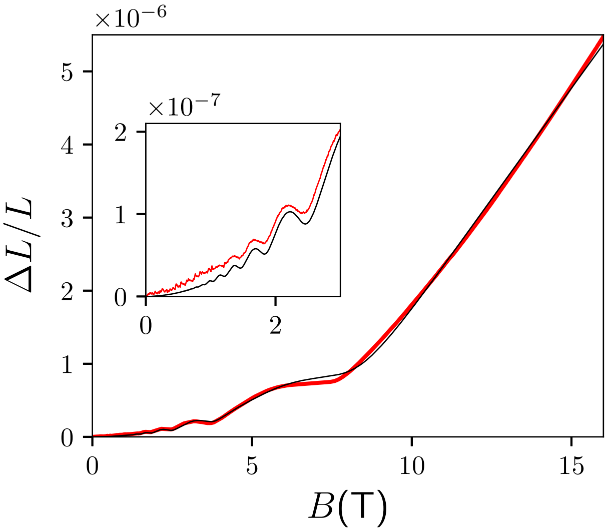

Supplementary Fig. 6. Comparison of the calculated magnetostriction with the experimental data for TaAs. The red line shows the c-axis magnetostriction $\Delta L/L$ of the crystal TaAs measured at T = 25 mK and $H \parallel c$ (see Fig. 2a in the main text). The black line depicts the magnetostriction calculated with equations (30), (32) [i.e. taking into account the B dependence of ζ] at zero temperature and at the values of the parameters presented in Supplementary Table I (set 1).

Supplementary Table I. **The values of the parameters for the calculation of the magnetostriction $\Delta L/L$ along the c axis and of the dependence $\zeta(B)$ at H aligned with c axis and at $v \equiv (\zeta_0 - \varepsilon_{d,W1})/(\zeta_0 - \varepsilon_{d,W2}) = 2.5$, $n_{W2}/n_{W1} = 0.15$.**

#	F_{W1} T	F_{W2} T	$\tilde\nu_h$	$10^3\tilde\beta_0$ T^{-2}	$10^3\tilde\beta_1$ T^{-2}	A_{W1} 10^{-6}	A_{W2} 10^{-6}	γ_{W1}	γ_{W2}
1	7.2	5	−0.91	1.63	1.53	−10.9	−1.92	0.025	0.1
2	7.2	1.35	−1.39	5.93	1.34	−4.72	−0.43	0.025	0.1

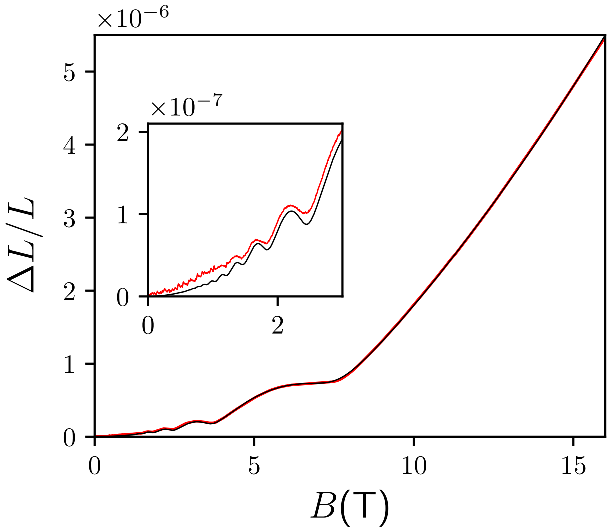

Supplementary Fig. 7. Comparison of the calculated magnetostriction with the experimental data for TaAs. The red line shows the c-axis magnetostriction $\Delta L/L$ of the crystal TaAs measured at $T = 25$ mK and $H \parallel c$ (see Fig. 2a in the main text). The figure is similar to Supplementary Fig. 6. In particular, the black line depicts the magnetostriction calculated with equations (30), (32) at zero temperature but with the other values of the parameters (set 2 in Supplementary Table I).

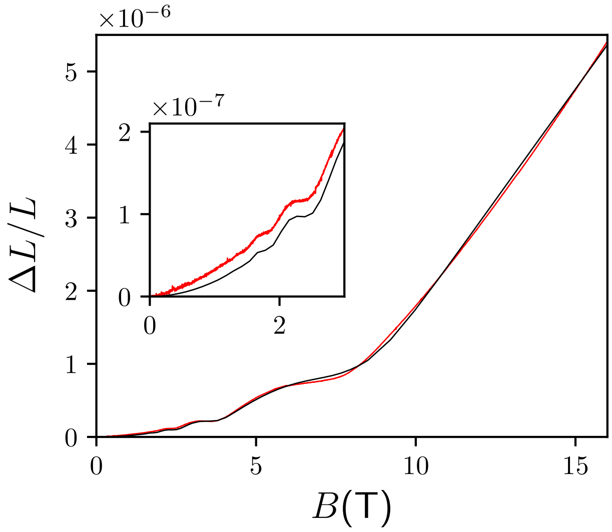

Supplementary Fig. 8. Comparison of the calculated magnetostriction with the experimental data at $T = 4.2$ K for TaAs. The red line shows the magnetostriction $\Delta L/L$ measured along the c axis at $T = 4.2$ K and $H \parallel c$ (see Fig. 2b in the main text). The black line corresponds to the magnetostriction calculated with Eqs. (30), (32), (33), using the first set of the parameters in Supplementary Table I and the dimensionless temperature $t = T/(\zeta_0 - \varepsilon_{W1}) = 0.015$. The inset is a zoom into the low-field region.

$(\zeta_0 - \varepsilon_{W2})\gamma_{W2}/\pi \sim 3.6$ K since the F_{W2} oscillations are unobservable in experiments, and γ_{W2} is not known precisely. In our calculations, we have set $\gamma_{W2} = 0.1$ only in order to suppress the oscillations with the frequency F_{W2}. Nevertheless, this value of $T_{D,W2}$ is reasonable. Assuming that the W1 and W2 electrons have approximately the same mean-free path $l_{\rm fp}$ and taking into account that $V_{W2}/V_{W1} \sim (16.52/1.92)^{1/3} \approx 2$ [24] where V_{Wi} are the mean velocities of the electrons in these pockets, we obtain $T_{D,W2} \sim 4.4$ K from $l_{\rm fp} \sim (\hbar/T_{D,W1})V_{W1} \sim (\hbar/T_{D,W2})V_{W2}$. Note also that a reduction of $T_{D,W2}$ to 2.5 K only marginally affects the agreement between the experimental and calculated data for the first set of the parameters and has no effect at all on the agreement for the second set when F_{W2} is small. Apart from $T_{D,W2} > T_{D,W1}$, the smallness of F_{W2} may be another reason why this frequency is not detected in experiments.

Analysis of the results. Knowing n_{W1}, n_{W2}/n_{W1}, and the values of the parameters $a_{W1} = -\Lambda^c_{W1} n_{W1}$, $a_{W2} = -\Lambda^c_{W2} n_{W2}$ obtained in the neglect of the B dependence of the chemical potential (see Supplementary Note 2 and Supplementary Table II), we can find the constants Λ^c_{W1} and Λ^c_{W2}, Supplementary Table II. Let us now estimate these constants in the case when the dependence of the chemical potential on B is taken into account. The coefficient $c_h = 1.49 \times 10^{-8}$ found in the neglect of this dependence can be written as $c_h = -\Lambda^c_h n_{W1} \tilde{\beta}_0$ (see the definitions of $\tilde{\beta}_0$ and β above). This relationship immediately gives Λ^c_h with the data of Supplementary Table I and $n_{W1} \approx 2.5 \times 10^{18}$. Then, the constants Λ^c_{W1} and Λ^c_{W2} can be calculated from the definitions $A_{W1} = -(\Lambda^c_{W1} - \Lambda^c_h)n_{W1}$ and $A_{W2} = -(\Lambda^c_{W2} - \Lambda^c_h)n_{W2}$. The results of these calculations for both the sets of the parameters are presented in Supplementary Table II. A comparison of the obtained constants Λ^c_i shows that in describing the magnetostriction, the use of the simplified approach, for which the B dependence of ζ is neglected, is reasonably well justified. (A caution should be given only to the case when one the parameters a_{Wi} is unusually small.) The same conclusion follows either from a comparison of Supplementary Figs. 5 and 6 or from the calculation of the dependence $\zeta(B)$ in the weak magnetic fields. Formula (30) for such fields yields $\tilde{\delta}\zeta \approx zB^2$ where the coefficient z is determined from the equation,

$$(3 + 3v\frac{n_{W2}}{n_{W1}} - \tilde{\nu}_h)z = -\frac{1}{16F^2_{W1}} + \frac{n_{W2}}{n_{W1}}\frac{1}{16F^2_{W2}} - \tilde{\beta}_0.$$

With the data of Supplementary Table I, we find that this coefficient is equal to $z_1 \approx 10^{-5}$ for the first set and to $z_2 \approx -7.6 \times 10^{-5}$ for the second one. On the other hand, in the weak-field range, formula (32) for the

Supplementary Table II. **The values of a_{Wi} and Λ_i^c obtained with and without the neglect of the dependence $\zeta(B)$.**

set #	$\zeta=\zeta(B)$	Λ^c_{W1} 10^{-24}cm^3	Λ^c_{W2} 10^{-24}cm^3	Λ^c_h 10^{-24}cm^3	a_{W1} 10^{-6}	a_{W2} 10^{-6}
1	−	0.63	1.6		−1.58	−0.60
1	+	0.71	1.47	−3.7		
2	−	0.85	0.17		−2.12	−0.062
2	+	0.89	1.42	−1.0		

magnetostriction reduces to the expression,

$$\frac{\Delta L}{L}=-A_{W1}\left(3\delta\tilde{\zeta}+\frac{B^2}{16F_{W1}^2}\right)-A_{W2}\left(3v\delta\tilde{\zeta}+\frac{B^2}{16F_{W2}^2}\right).$$

Comparing, e.g., the terms $3\delta\tilde{\zeta}$ and $B^2/(16F_{W1}^2)$, one obtains that the first term is smaller than the second one by the factor 40 for set 1 and by the factor 5 for set 2. In other words, in the first approximation, one really can neglect the dependence $\zeta(B)$.

Assuming the parabolic spectrum of the holes and inserting the data of Supplementary Table I for set 1 into formulas (31), we obtain that $\tilde{\beta}_1/\tilde{\beta}_0\approx 0.94$ and $|n_h|/n_{W1}\approx 0.32$. The ratio $\tilde{\beta}_1/\tilde{\beta}_0$ agrees with the estimate derived from the data of Ref. [18] (Supplementary Note 6). The obtained value of $|n_h|/n_{W1}$ means that the doping in our sample, $n_{W1}+n_{W2}-|n_h|$, is of the order of $0.83n_{W1}\approx 2\times10^{18}\,\mathrm{cm}^{-3}$. As to the value of $\tilde{\beta}_0$ presented in Supplementary Table I for set 1, it can be reproduced with the first formula of (31) at $|n_h|/n_{W1}=0.32$ and $F_h=19\,\mathrm{T}$ if we assume that due to the spin-orbit interaction, the magnetic moment of the holes is sufficiently large (the parameter δ amounts to 2.2).

A similar analysis for the second set of the parameters in Supplementary Table I leads to the unrealistic value of $\varepsilon_0-\zeta_0\approx 63$ meV which essentially exceeds the estimate $\varepsilon_0-\zeta_0\approx 12.6\div16.2\,\mathrm{meV}$ inferred from the results of Arnold et al. [18] under the assumption of the parabolic spectrum for the holes (Supplementary Note 6). Moreover, formulas (31) with set 2 give $|n_h|/n_{W1}\approx 2.05$, the value of which disagrees with the electron doping of the specimen that was used in Ref. [18] and that is similar to ours. These discrepancies indicate that the second set of the parameters may be admissible only if a significant deviation of the dispersion of the holes from the parabolic law occurs and hence if formulas (31) and the estimate for $\varepsilon_0-\zeta_0$ in Supplementary Note 6 are not applicable to this charge carriers.

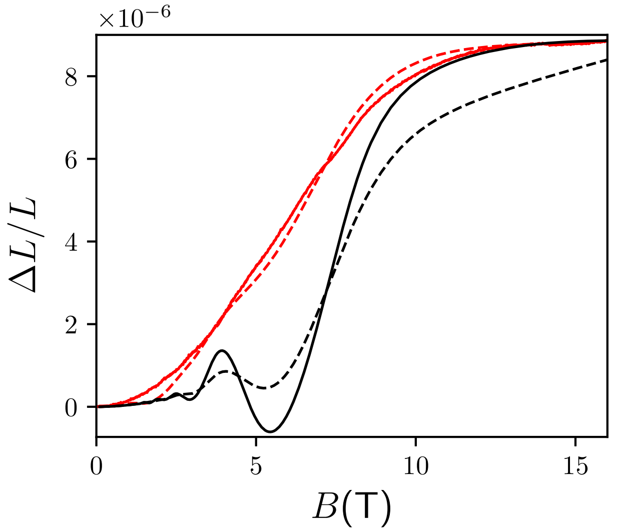

Supplementary Fig. 9. Comparison of the calculated magnetostriction with the experimental data for TaAs. The solid red line shows the relative length change $\Delta L/L$ of TaAs (sample 2) measured along the [100] direction at 25 mK for the magnetic field aligned with the c axis. The dashed red line depicts the a-axis magnetostriction calculated at $T=0$, using set 2 of the parameters in Supplementary Table I, but with new $A_{W1}=15.7\times10^{-6}$, $A_{W2}=-2.61\times10^{-6}$, $\gamma_{W1}=0.1$, and $\gamma_{W2}=0.2$. The solid black line corresponds to set 1, but with $A_{W1}=1.48\times10^{-6}$, $A_{W2}=-17.2\times10^{-6}$, $\gamma_{W1}=0.06$, and $\gamma_{W2}=0.1$. The dashed black line is plotted for the same parameters as the solid black curve except for $\gamma_{W1}=0.1$, and $\gamma_{W2}=0.2$.

Supplementary Note 8: Magnetostriction along the a axis

Consider the magnetostriction $\Delta L/L$ along the a axis for the magnetic field still aligned with the direction [001]. In this case, in describing the magnetostriction, only the values of A_{W1} and A_{W2} can change due to a change of the constants Λ_{W1}, Λ_{W2}, and Λ_h. All the other parameters determining this $\Delta L/L$ should remain the same as in the case of the c-axis magnetostriction. Therefore, a comparison of theoretical and experimental results for the a-axis magnetostriction may enable one to choose between the two sets of the parameters presented in Supplementary Table I.

The magnetostriction $\Delta L/L$ measured along the a axis at B parallel to [001] is shown in Supplementary Fig. 9. In this figure, we also show the magnetostriction calculated with both the sets of the parameters in Supplementary Table I, varying only the constants A_{W1} and A_{W2}. (We also use modified values of γ_{W1} and γ_{W2} in order to suppress the oscillations; note that Supplementary Figs. 6-8 and 9 show the magnetostrictions for the two different samples). For the first set, we are not able

Supplementary Table III. **The values of Λ_i^c, $\Lambda_i^\perp$, $\lambda_{zz}^{(i)}$, and $\bar{\lambda}_{xx}^{(i)}$ for the second set of the parameters in Supplementary Table I (i=W1, W2, h).**

	Λ_i^c 10^{-24}cm^3	$\Lambda_i^\perp$ 10^{-24}cm^3	$\lambda_{zz}^{(i)}$ eV	$\bar{\lambda}_{xx}^{(i)}$ eV
W1	0.89	−2.1	2.2	5.7
W2	1.42	11.2	−21.7	−35.5
h	−1.0	4.2	−5.6	−12

to match well the theoretical curve with the experimental data. The solid black line shows the best result that we have been able to obtain. An increase of γ_{Wi} even enhances the disagreement (the dashed black line), and a subsequent variation of A_{Wi} does not improve this situation noticeably. For the second set, the obtained theoretical curve approximately reproduces these data if we take $A_{W1} = 15.7\times 10^{-6}$ and $A_{W2} = -2.61\times 10^{-6}$ together with the modified values of $\gamma_{W1} = 0.1$ and $\gamma_{W2} = 0.2$. Since the parameter $\lambda \equiv (1/L)d\Delta L/dB$ in the ultra-quantum region $B \gtrsim 8$ T has the form $2c_hB + b$, the slope of this linear dependence gives the parameter $2c_h$ (see Supplementary Fig. 3). Similarly to Supplementary Note 7, with the obtained A_{W1}, A_{W2}, $c_h \approx -6.25\times 10^{-8}$, and with set 2 from Supplementary Table I, we find the constants $\Lambda_{W1}^\perp$, $\Lambda_{W2}^\perp$, and $\Lambda_h^\perp$ defined in Supplementary Note 1 (Supplementary Table III). Knowing these constants $\Lambda_i^\perp$ and the constants Λ_i^c (set 2 in Supplementary Table II), one can calculate $\lambda_{zz}^{(i)}$ and $\bar{\lambda}_{xx}^{(i)}$ from Eqs. (5); Supplementary Table III. Note that the values of $\lambda_{zz}^{(i)}$ and $\bar{\lambda}_{xx}^{(i)}$ make it possible to predict how the frequencies of quantum oscillations in magnetic fields for the W1 and W2 electrons and for the holes change under a uniform compression. It is clear from the data of Supplementary Table III that the Fermi surface of the W2 electrons should be most sensitive to this compression.

The presented fit of the theoretical curve to the experimental data argues in favor of set 2. However, we emphasize that the orientation of the sample in respect to the magnetic field direction cannot be established in our experiments with a high accuracy, and hence the experimental curve presented in Supplementary Fig. 9 does not necessarily corresponds to the case of $B \parallel [001]$. Due to the high sensitivity of the a-axis magnetostriction to the angle between B and the c axis (see Fig. 6 in the main text), its true B dependence at $B \parallel [001]$ may essentially differ from the experimental curve presented in Supplementary Fig. 9, and so a more precise orientation of the sample is required in order to reliably exclude the possibility of set 1.

Let us now discuss the unusual high sensitivity of the a-axis magnetostriction to small deviation of B from the c axis (Fig. 6 in the main text). When the magnetic field is tilted away from the direction [001] in the plane (010), electron pockets in the W1, W2 groups, as well as the hole pockets, produce different contributions to the magnetostriction, and one should take into account that with increasing the tilt, the parameters F_{W1}, F_{W2}, F_h not only change in magnitude but also the number of these parameters increases (the charge-carrier pockets in any group have different values of these parameters). This makes the strict theoretical analysis of the magnetostriction very complicated. However, the low sensitivity of the c-axis magnetostriction to the deviation angle (Fig. 7 in the main text) indicates that the total differences of the densities $n(B) - n(0)$ for each of the groups (W1, W2, h) change only slightly with the angle. As is explained in Supplementary Note 1, the a-axis magnetostriction contains an additional term that is proportional to $n_{a1}(B) - n_{a2}(B)$ where n_{a1} and n_{a2} are the charge-carrier densities of the equivalent pockets lying near the reflection planes (010) and (100), respectively. Consider the situation when equivalent pockets have the shape of elongated ellipsoids, the longest axes of which are practically perpendicular to the c axis. This model seems to be applicable to the Fermi surface of the holes in TaAs. When the magnetic field is tilted in the (010) plane, the appropriate extremal cross-sectional areas of the ellipsoids lying near this plane abruptly decreases, whereas the appropriate areas for the ellipsoids located near the (100) plane remain practically unchanged. This means that in the weak magnetic fields, the difference $|n_{a1}(B) - n_{a2}(B)|$ steeply increases with the tilt angle. Calculating $\lambda(B) \equiv (1/L)d\Delta L/dB$ for the curves shown in Fig. 6 of the main text and approximating these $\lambda(B)$ by $2c_hB + b$ at $B > 8$ T, we find the following values of $c_h(\Theta)$ (in units of 10^{-8} T^{-2}):

$$c_h(90^\circ) \approx -6.25, \quad c_h(87^\circ) \approx 0,$$
$$c_h(86^\circ) \approx 4.5, \quad c_h(81^\circ) \approx 3.8,$$

which do indicate the large variation of c_h with the angle Θ between B and the a axis. This sharp variation may be due to the above-mentioned strong Θ dependence of $|n_{a1}(B) - n_{a2}(B)|$ for the holes. We cannot also exclude that the Fermi-surface pockets for the W2 electrons have a shape other than spherical, and so they can contribute to the angular dependence of the a-axis magnetostriction as well. (Although this assumption does not agree with the numerical calculation of the Fermi surface for the W2 electrons [18], however, subtle details of this small surface can hardly be obtained in the calculations.) On the other hand, the W1 pockets have the shape close to ellipsoids elongated in the [001] direction, and so they give a gradually varying contribution to this angular dependence.

[1] Küchler, R., Bauer, T., Brando, M. & Steglich, F. A compact and miniaturized high resolution capacitance dilatometer for measuring thermal expansion and magnetostriction. *Rev. Sci. Instrum.* **83**, 095102 (2012).
[2] Landau, L.D. & Lifshitz, E.M. *Theory of Elasticity* (Pergamon Press, 1981).
[3] Mikitik, G.P. & Sharlai, Yu.V. Specific features of magnetostriction at electron topological transitions in metals. *Low Temp. Phys.* **43**, 168-172 (2017).
[4] Keyes, R. W. Electronic Effects in the Elastic Properties of Semiconductors. *Solid State Phys.* **20**, 37-90 (1968).
[5] Michenaud, J-P., Heremans, J., Shayegan, M. & Haumont, C. Magnetostriction of bismuth in quantizing magnetic fields. *Phys. Rev. B* **26**, 2552-2559 (1982).
[6] Mikitik, G. P. & Sharlai, Yu. V. Spontaneous symmetry breaking of magnetostriction in metals with multivalley band structure. *Phys. Rev. B* **91**, 075111 (2015).
[7] dos Reis, R. D. et al. Pressure tunning the Fermi surface topology of the Weyl semimetal NbP. *Phys. Rev. B* **93**, 205102 (2016).
[8] Abrikosov, A.A. *Fundumentals of the theory of metals* (North-Holland, Amsterdam, 1988).
[9] Buckeridge, J., Jevdokimovs, D., Catlow, C.R.A. & Sokol, A.A. *Phys. Rev. B* **93**, 125205 (2016).
[10] Mikitik, G. P. & Sharlai, Yu. V. Magnetic Susceptibility of Topological Semimetals. *J. Low Temp. Phys.* **197**, 272-309 (2019).
[11] Mikitik, G.P. & Sharlai, Yu. V. Field dependences of magnetic susceptibility of crystals under conditions of degeneracy of their electron energy bands. *Fiz. Nizk. Temp.* **22**, 762-771 (1996) [*Low Temp. Phys.* **22**, 585-592 (1996)]. (See the full text at https://www.researchgate.net/profile/G-Mikitik/2)
[12] Bateman, H. & Erdelyi, A. *Higher transcendental functions Vol. 1, Ch. 1.10 and 1.18* (Mc Graw-Hill Book Company, Inc, New York, Toronto, London, 1953).
[13] Shoenberg, D. *Magnetic Oscillations in Metals* (Cambridge University Press, Cambridge, England, 1984).
[14] Mikitik, G. P. & Sharlai, Yu. V. Manifestation of Berry's phase in metal physics. *Phys. Rev. Lett.* **82**, 2147-2150 (1999).
[15] Mikitik, G. P. & Sharlai, Yu. V. g factor of conduction electrons in the de Haas−van Alphen effect. *Phys. Rev. B* **65**, 184426 (2002).
[16] Mikitik, G. P. & Sharlai, Yu. V. Calculation of conduction electron g factor in metals: Comparison of electron-spin dynamics and local g-factor approaches. *Phys. Rev. B* **67**, 115114 (2003).
[17] Küchler, R. et al. Thermodynamic evidence for valley-dependent density of states in bulk bismuth. *Nat. Mater.* **13**, 461-465 (2014).
[18] Arnold, F. et al. Chiral Weyl pockets and Fermi surface topology of the Weyl semimetal TaAs. *Phys. Rev. Lett.* **117**, 146401 (2016).
[19] Moll, P. J. W. et al. Magnetic torque anomaly in the quantum limit of Weyl semimetals. *Nat. Commun.* **7**: 12492 (2016).
[20] Zhang, C.-L. et al. Non-saturating quantum magnetization in Weyl semimetal TaAs. *Nat. Commun.* **10**: 1028 (2019).
[21] Modic, K.A. et al. Thermodynamic signatures of Weyl fermions in NbP. *Sci. Rep.* **9**: 2095 (2019).
[22] Mikitik, G. P. & Sharlai, Yu. V. Magnetic susceptibility of topological nodal semimetals. *Phys. Rev. B* **94**, 195123 (2016).
[23] Mikitik, G. P. & Sharlai, Yu. V. Analysis of Dirac and Weyl points in topological semimetals via oscillation effects. *Low Temp. Phys.* **47**, 312-317 (2021).
[24] Grassano, D., Pulci, O., Cannussia, and Bechstedt, F., Influence of anisotropy, tilt and pairing of Weyl nodes: the Weyl semimetals TaAs, TaP, NbAs and NbP. *Eur. Phys. J. B* **93**: 157 (2020).
